# Case Report: Two Infant Cases of Langerhans Cell Histiocytosis Involving the Digestive Tract

**DOI:** 10.3389/fped.2021.545771

**Published:** 2021-02-24

**Authors:** Huan Wang, Yuesheng Wang, Ruifeng Wang, Xiaoqin Li

**Affiliations:** Department of Gastroenterology, The Affiliated Children Hospital of Zhengzhou University, Henan Children Hospital, Zhengzhou, China

**Keywords:** langerhans cell histiocytosis, gastrointestinal tract, infant, endoscopic manifestation, BRAF mutation (V600E)

## Abstract

Langerhans cell histiocytosis (LCH) is a rare disease with uncertain etiology. Langerhans cell histiocytosis with involvement of the gastrointestinal tract is rare and is typically identified in pediatric patients with systemic disease. The present study reports two infantile cases of LCH who initially presented with diarrhea, hematochezia, and rash and were histologically missed on the original examination of the colonic biopsy sections. The diagnosis of LCH was later verified through immunohistochemistry. By combining our experience and previous reports, the multiple hemorrhagic spots of the colorectal mucosa and narrowness and erosion of the distal duodenum might be suggestive manifestations of gastrointestinal involvement in LCH on endoscopic examination. This might be helpful for the early recognition of the disease.

## Introduction

Langerhans cell histiocytosis (LCH) is a rare disease with uncertain etiology. It is characterized by the abnormal proliferation and dissemination of Langerhans cells derived from the bone marrow. The peak incidence occurs in children between 1 and 5 years of age. Allen et al. (1) reported that the incidence in children was ~5 in 1 million. Langerhans cell histiocytosis with involvement of the gastrointestinal tract (GIT) is rare, and is typically identified in pediatric patients with systemic disease who carry a poor prognosis (2). Langerhans cell histiocytosis can involve any part of the digestive tract, with the most common being the duodenum and colon (3). The clinical symptoms vary from refractory diarrhea and bloody stool to vomiting and hypoproteinemia (4). Here, we report two infants who presented with refractory diarrhea and hematochezia and were diagnosed with LCH based on specific endoscopic manifestations, histopathology, and immunohistochemistry.

## Case Presentation

### Case 1

**Figure 1:**
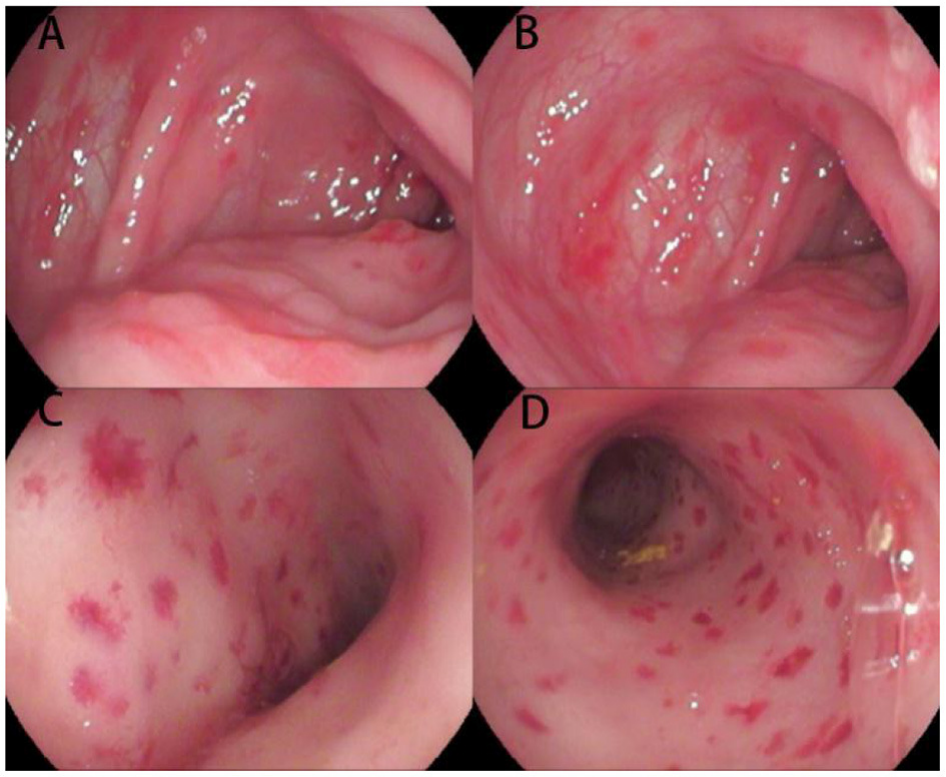
Endoscopic images of the colon and rectum for Case 1. Multiple hemorrhagic spots, erosions, and ulcerations of the mucosa are present from the ascending colon **(A,B)** to the rectum **(C,D)**.

A 10-month-old female was transferred to our hospital for recurrent diarrhea (intermittently mixed with blood) and failure to gain weight for 3 months. The patient was diagnosed as “allergic enteropathy with suspicion of ulcerative colitis” based on initial colonoscopy findings. Treatment for 1 month with amino acid–based milk and mesalazine (10 mg/kg, three times a day) produced limited effects. On physical examination, the patient's weight was 7.4 kg (~20th percentile of WHO standards). Scattered new and old red papules with desquamation were present on the back. No organomegaly or perianal lesions were noted. The mother gave a history of recurrent red papules on the back beginning 1 month before without any treatment. The initial laboratory investigation revealed leukocytosis with a white blood cell count of 16.5 K/uL (normal, 6.0–14.0), and thrombocytosis with a platelet count of 804 K/uL (normal, 150–450). The immunoglobulin level, liver and renal function, lactate dehydrogenase, C-reactive protein (CRP), erythrocyte sedimentation rate (ESR), and T-SPOT.TB were normal. Serology for Epstein Barr, cytomegalovirus, hepatitis-B, hepatitis-C, and human immunodeficiency viruses was negative, as were tests for food allergies, the presence of clostridium difficile, and the stool culture. The possible diagnosis of inflammatory bowel disease was considered and intravenous methylprednisolone (1 mg/kg every 12 h for 5 days) was given, which was ineffective. Then, a colonoscopy was performed. This revealed multiple hemorrhagic spots, erosions, and ulcerations of the mucosa from the ascending colon to the rectum (Figure 1). Histopathology of the biopsy revealed infiltration of intermediate-sized mononuclear cells with abundant cytoplasm and convoluted nuclei. Many of these cells in the colorectal mucosa had linear grooves consistent with Langerhans cells, accompanied by eosinophil infiltration. Immunohistochemistry for CD1a, CD68, CD163, Langerin, and S-100 was positive, consistent with Langerhans cells. The skeletal survey, chest and abdominal computed tomography, cranial magnetic resonance imaging, and bone marrow aspiration test were negative.

Based on LCH criteria (5), the patient was diagnosed with multi-system LCH involving the GIT and skin. The patient received weekly chemotherapy with 6 mg/m^2^ vinblastine and daily oral prednisolone (40 mg/m^2^) for 6 weeks, at which time the patient was asymptomatic. Then, the patient received treatment according to the LCH-III protocol. In the subsequent 2 years, no diarrhea or hematochezia were observed and the weight percentile increased.

### Case 2

A 5-month 24-day-old female infant presented with poor weight gain, recurrent diarrhea, and hematochezia for 2 months. The patient was initially considered to have infectious diarrhea, but the corresponding treatment was ineffective. Hypoalbuminemia (albumin, 1.67 g/dL) was noted 12 days prior to admission.

On physical examination, the patient's weight was 5.9 kg (~10th percentile of WHO standards). Red papules were observed on the geisoma, scalp, chest, and back, with erosion and scabbing. Redness, erosion, and desquamation were observed in the wrinkle parts of the neck and groin skin, as well as ecchymosis in the perineum. Obvious erosion and inflammatory granulation of the perianal skin were observed. The mother recalled that the rash erupted initially after birth and was treated as a seborrheic or eczematoid rash, with limited effect.

After admission, laboratory investigations revealed anemia (hemoglobin 6.7 g/dL) and hypoalbuminemia (albumin: 1.7 g/dL). The immune status, ESR,CRP, blood coagulation, blood ammonia, and T-SPOT.TB tests were normal. Serology for Epstein Barr, cytomegalovirus, rotavirus, adenovirus, hepatitis-B, hepatitis-C, and human immunodeficiency viruses were negative, as was the test for clostridium difficile and the stool culture. The bone marrow aspiration test was normal. A computed tomography scan of the lung revealed patchy shadows.

**Figure 2:**
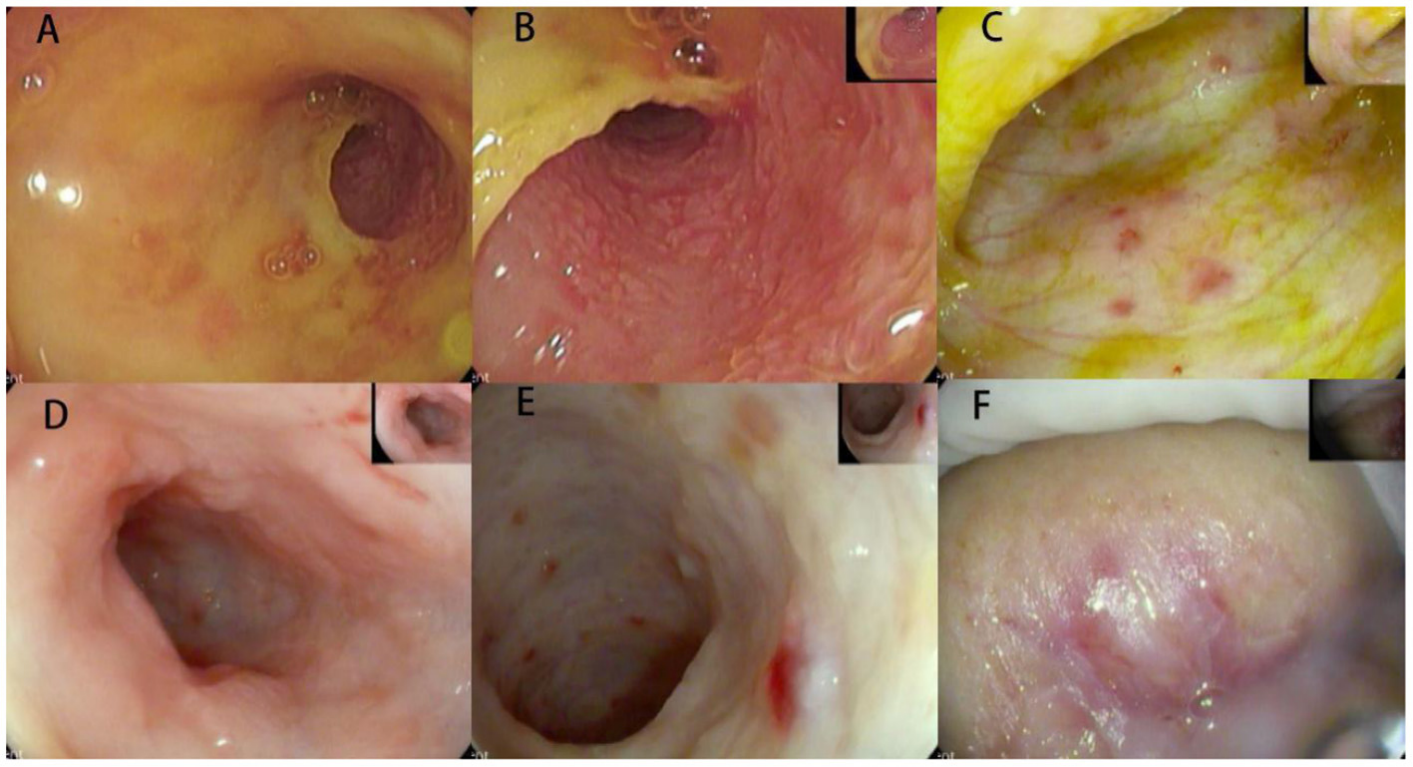
Endoscopic images for Case 2. **(A,B)** Mucosal swelling and erosions of the mucosa, as well as narrowness and erosion of the distal duodenum beyond which the scope could not pass, area evident. **(C–E)** Hemorrhagic spots, nodules of the colonic mucosa, and disappearance of the colon band in the colon mucosa. **(F)** Inflammatory granulation of the perianal area.

**Figure 3:**
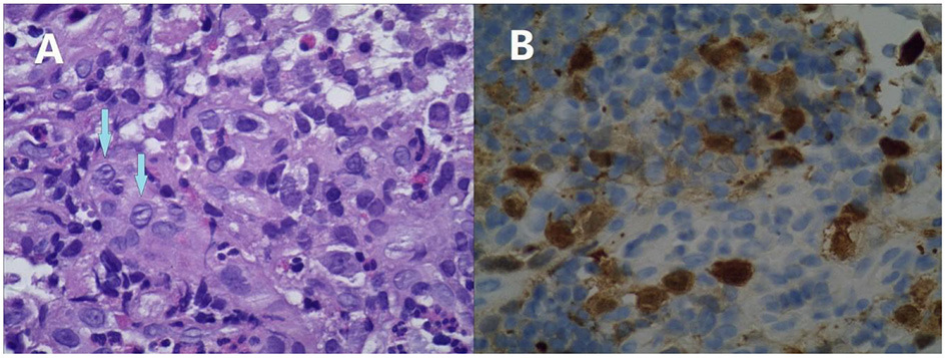
Microphotographs of the colonic biopsy of Case 2. Infiltration of mononuclear cells with an abundant cytoplasm and convoluted nuclei. Many of these cells have linear grooves that are consistent with Langerhans cells (arrows, **A**). These are accompanied by mixed inflammatory cell infiltrates, including eosinophils (original magnification ×400). These cells are immunoreactive for CD1a **(B)**.

Protein losing enteropathy was suspected and albumin supplementation was given. Upper and lower endoscopies were performed to search for causes. The upper endoscopy revealed narrowness and erosion of the distal duodenum, through which the 7.5-mm diameter endoscope was unable to pass. The colonoscopy revealed multiple hemorrhages and edema of the colonic mucosa (Figure 2). Pathologic evaluation of the biopsies revealed no specific findings. The skin punch biopsy showed histiocytes with features consistent with Langerhans cells in the upper dermis. The colonic biopsy was reviewed retrospectively. This revealed infiltration of large atypical cells with grooved nuclei and an abundant eosinophilic cytoplasm in the ileocecal, descending colon, and rectum mucosa, which stained positive for CD1a and S-100 (Figure 3). The skeletal survey, abdominal computed tomography, and cranial magnetic resonance imaging were negative.

Based on the GIT findings, and skin, and lung involvement, the patient was diagnosed with LCH (multiple system involvement). The BRAF mutation test revealed a BRAF V600E mutation. A standard chemotherapy protocol consisting of an initial intensive phase for 12 weeks, followed by maintenance for a total treatment duration of 8 months, was administered. At that time, thrombocytopenia and skull bone damage emerged, and recurrent diarrhea and hematochezia were observed. Hence, the targeted BRAF(V600) medicine, dabrafenib, was administered orally, after which the patient became stable.

## Discussion

Involvement of the GIT in LCH children is rare. Such children have a poor prognosis and high rates of morbidity, and usually present with the involvement of multiple systems. Over 50% of LCH patients die within 18 months from diagnosis (3). A retrospective study conducted by Yoon et al. (6) revealed that LCH with GIT involvement was associated with a 4-fold increased risk for organ involvement, which correlated with increased mortality. Therefore, early recognition and timely treatment are of great importance. In the present study, we report two infant LCH cases with symptoms of diarrhea and hematochezia. These symptoms, along with atypical histopathological findings, such as the infiltration of inflammatory cells (1), could be easily misdiagnosed as protein loss enteropathy, allergic or infectious enteritis, an immunodeficiency disease, or irritable bowel disease (7, 8).

It is noteworthy that these two cases had some specific endoscopic manifestations. In Case 1, hemorrhagic spots were observed, which is rarely seen in pediatric patients but was reported in a 6-month-old female (9). As suggested by the pathologists, immunohistochemistry was performed and LCH was verified. Our experience is similar to that reported by Badr et al. (10). This suggests that multiple hemorrhagic spots of the colorectal mucosa might be a suggestive manifestation of GIT involvement in LCH, though this needs to be verified through studies with a larger sample size. In Case 2, narrowness and erosion of the distal duodenum were observed in the upper endoscopy. Markus et al. reported a 16-month-old female with similar endoscopic manifestations who was diagnosed as LCH (11). Indeed, several studies presented similar findings for LCH (8–12), suggesting that narrowness of the distal duodenum might be a specific feature of LCH-GIT. Furthermore, the rash in the present patient was chronic and recurrent. Except for atypical papules and eczematous rash, the patient presented with refractory scaly, scabbing papules of the scalp and ecchymosis of the perineum. These manifestations should be considered for the differential diagnosis of LCH, and a skin biopsy taken.

The pathogenesis of LCH remains uncertain. Recent research has shown that activating mutations in mitogen-activated protein kinase pathway genes occur in most LCH cases, and ~60% were attributable to BRAF-V600E (13). The BRAF kinase inhibitor, dabrafenib, has a therapeutic effect in a subset of these patients, and has been recommended for treating refractory and severe cases with a BRAF V600E mutation (14). Consistent with this, for Case 2, after the administration of dabrafenib, the patient became stable.

In conclusion, involvement of the GIT in LCH is rare and could be easily overlooked. For infant patients with refractory diarrhea and hematochezia, especially with an atypical rash, LCH-GIT should be considered. Furthermore, similar to our previous experience and reports, multiple hemorrhagic spots of the colorectal mucosa, as well as narrowness and erosion of the distal duodenum, might be suggestive manifestations of LCH-GIT in the endoscopy tests.

## Data Availability Statement

The original contributions generated in the study are included in the article/supplementary material, further inquiries can be directed to the corresponding author.

## Ethics Statement

Written informed consent was obtained from the individual(s), and minor(s)' legal guardian/next of kin, for the publication of any potentially identifiable images or data included in this article.

## Author Contributions

HW and XL drafted the manuscript. RW and YW acquired, analyzed, and interpreted the data. XL edited the manuscript. All authors contributed to the article and approved the submitted version.

## Conflict of Interest

The authors declare that the research was conducted in the absence of any commercial or financial relationships that could be construed as a potential conflict of interest.
